# Modular assembly of a protein nanotriangle using orthogonally interacting coiled coils

**DOI:** 10.1038/s41598-017-10918-6

**Published:** 2017-09-05

**Authors:** Won Min Park, Mostafa Bedewy, Karl K. Berggren, Amy E. Keating

**Affiliations:** 1 Department of Biology, Massachusetts Institute of Technology, 77 Massachusetts Avenue, Cambridge, Massachusetts 02139 USA; 2 Research Laboratory of Electronics, Massachusetts Institute of Technology, 77 Massachusetts Avenue, Cambridge, Massachusetts 02139 USA; 3 Department of Electrical Engineering and Computer Science, Massachusetts Institute of Technology, 77 Massachusetts Avenue, Cambridge, Massachusetts 02139 USA; 4 Department of Biological Engineering, Massachusetts Institute of Technology, 77 Massachusetts Avenue, Cambridge, Massachusetts 02139 USA; 5 Present Address: Department of Industrial Engineering, University of Pittsburgh, 3700 O’Hara Street, Pittsburgh, Pennsylvania 15261 USA

## Abstract

Synthetic protein assemblies that adopt programmed shapes would support many applications in nanotechnology. We used a rational design approach that exploits the modularity of orthogonally interacting coiled coils to create a self-assembled protein nanotriangle. Coiled coils have frequently been used to construct nanoassemblies and materials, but rarely with successful prior specification of the resulting structure. We designed a heterotrimer from three pairs of heterodimeric coiled coils that mediate specific interactions while avoiding undesired crosstalk. Non-associating pairs of coiled-coil units were strategically fused to generate three chains that were predicted to preferentially form the heterotrimer, and a rational annealing process led to the desired oligomer. Extensive biophysical characterization and modeling support the formation of a molecular triangle, which is a shape distinct from naturally occurring supramolecular nanostructures. Our approach can be extended to design more complex nanostructures using additional coiled-coil modules, other protein parts, or templated surfaces.

## Introduction

Protein assembly is a versatile bottom-up approach for creating precise structures at the nanometer scale^1^. Such structures are critical to the development of novel materials and biologically functional devices. Short peptides provide minimal units for non-covalent association to mediate self-assembly, and substantial advances have been achieved using naturally derived or synthetic peptides to make fibers, nanotubes and mesh-like scaffolds^2^. Folded protein assembly is guided by shape- and chemical-complementarity that can establish highly specific non-covalent associations. Protein-protein interfaces can be harnessed or newly designed to build geometrically well-defined nanostructures, although the complex and cooperative interactions at interfaces are difficult to control rationally. Recently, computational methods have enabled stunning *de novo* design of protein nanostructures including two-dimensional arrays^3^ and cages with diverse structural features^4–7^. A rational approach to building with native components is to combine known folds into fused assembly units. Symmetric cages and cubes have been constructed in this way, with distinct domain interfaces aligned into controlled geometries by rigid helical linkers^8–10^. Similarly, protein assembly has been demonstrated using oligomeric protein domains connected by short linkers^11, 12^ biotin-streptavidin binding^13^, or metal-directed coordination^14^.

As an alternative to designing specialized components for each assembly task, a set of modular building blocks can potentially provide enhanced flexibility for constructing biomolecular nanostructures. For example, coiled coils, which are rod-like complexes of two or more supercoiled α-helices, have designable specificity, oligomeric state, length, and helix orientation. The interactions between helices in coiled coils is controlled by hydrophobic-polar and charge patterning of amino acids in a seven-residue repeat that is conventionally labeled *abcdefg*, with *a* and *d* positions typically hydrophobic^15, 16^. Based upon a rich understanding of coiled-coil sequence-structure relationships that has been obtained over the past ~25 years, *de novo* designed coiled-coil toolkits, i.e. sets of coiled-coil units with known interaction properties, have been generated by using systematic search and/or rational^17, 18^ or computational approaches^19–23^. Because of their utility as short self-assembling protein modules, coiled coils have been used to construct a variety of assemblies, including fibers^24^, cages^25, 26^, and nanotubes^27^ with extended and geometrically irregular structures^28^.

Modular design of coiled-coil assemblies with specific atomically definable three-dimensional structures^28^ has been challenging, and has only been demonstrated in few cases. In one striking example, multiple distinct coiled coils were arranged into a single chain that folds into a tetrahedron, with the final structure dictated by specific coiled-coil associations^29^. The single-chain design relied on a careful ordering of elements within the chain to control the topology, which may limit the broader utility of this approach. So far, no other single-chain folded shapes based on coiled-coil modules have been reported. A different method using coiled coils to create polygonal nanoscale objects was reported, in which linked coiled coils formed self-assembling structures^30^. Because only a single type of coiled-coil heterodimer was used in this work, control over the number of subunits was introduced using linker lengths, limiting modularity and potential generalizability.

**Figure 1:**
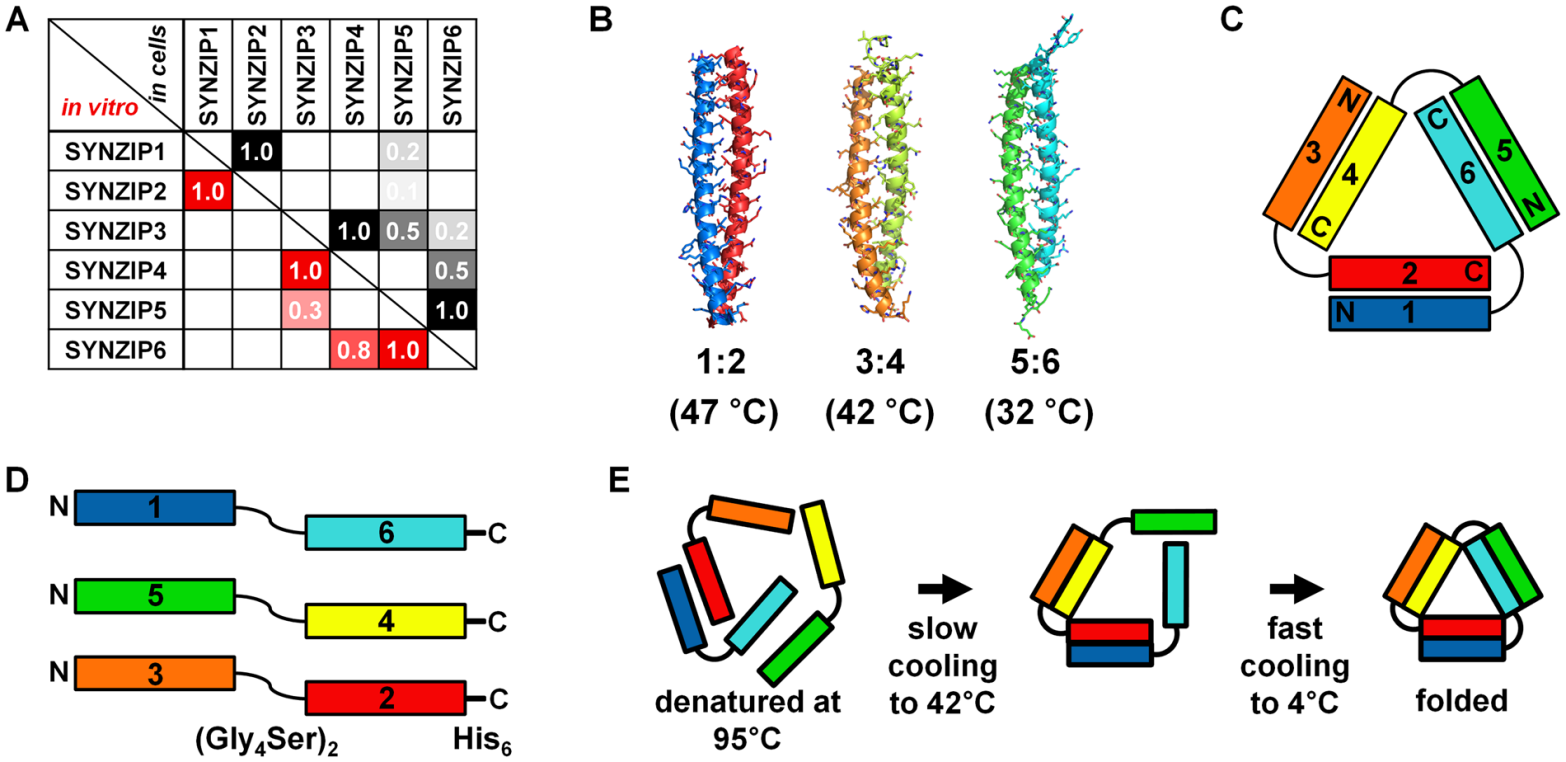
Orthogonally interacting SYNZIPs underlie the design of a protein nanotriangle. (**A**) Pairwise interaction profiles of SYNZIPs (**1** to **6**) determined by protein microarrays (*in vitro*, lower left)^20^ and the yeast two-hybrid assays (in cells, upper right)^19^. Assay scores were normalized to a scale where 1.0 indicates tight binding. The corresponding *K*_D_ values are <10, <30, and <15 nM for **1**:**2**, **3**:**4**, and **5**:**6**, respectively, and are approximated to be >400 nM for **3**:**5**, and **4**:**6** ^19^. (**B**) Crystal structures of **1**:**2** (PDB ID: 3HE5) and **5**:**6** (PDB ID: 3HE4)^20^, and a model of **3**:**4**; melting temperatures are given in parentheses. (**C**) Topology of the designed heterotrimeric protein nanotriangle composed of the orthogonal SYNZIPs. (**D**) The composition of individual linked-SYNZIP fusion proteins that self-assemble into the nanotriangle shown in panel C. (**E**) A two-step thermal annealing process for assembly of the linked-SYNZIP fusion proteins, and illustration of desired assemblies at each step.

Here, we report a simple and rational design of a protein nanotriangle that exploits the modularity of multiple orthogonally interacting coiled coils. The construction and characterization process involved: (1) design of appropriately linked coiled-coil modules; (2) recombinant production and purification of designed proteins; (3) mixing and annealing according to an optimized schedule; and (4) biophysical analysis of the assembled nanotriangle. Three previously characterized heterodimeric coiled-coil modules that specifically associate while avoiding undesired crosstalk were arranged into self-assembling building blocks (Fig. 1). The assembly geometry was encoded by the strategic fusion of pairs of non-associating sequences in combinations that disfavored competing assemblies. Specific dimerization of the designed modules favored formation of coiled-coil edges connected into a triangular shape via flexible linkers.

## Results

### Design of a self-assembled protein nanotriangle

Three orthogonally interacting pairs of coiled-coil modules were selected from 23 previously reported synthetic heterodimeric coiled coils (SYNZIPs) (Fig. 1A)^19–21^. The three SYNZIP pairs **1**:**2**, **3**:**4**, and **5**:**6** (where a colon indicates non-covalent interaction) interact with high affinity (assay scores ~1.0) and are favored over weak undesired crosstalk with other SYNZIPs (assay scores < 0.8). The dissociation constants for the three binding pairs (*K*_D_ < 30 nM) are at least an order of magnitude lower than those for any other pairwise interactions within this set of proteins (*K*_D_ > 400 nM)^19^. Crystal structures indicate that complexes **1**:**2** (PDB ID: 3HE5) and **5**:**6** (PDB ID: 3HE4) are parallel, heterodimeric coiled coils (Fig. 1B)^20^. Complex **3**:**4** is also a parallel heterodimer, and the axial sequence alignment has been determined experimentally^19, 20^. Based on these characterizations, we built a structural model for **3**:**4** by comparative modeling^31^ and refined it by molecular dynamics simulation for 10 ns^32^. The model shows interactions between polar and charged residues at *e* and *g* positions, and hydrogen bonding of asparagine residues at the *a*–*a*′ positions (Figs 1B and S1) that are characteristic of many parallel coiled-coil dimers^15^.

To design a protein nanotriangle, we explored all 16 possible ways that 6 SYNZIPs can be distributed in a three-chain topology (Fig. S2). Interactions between coiled coils that are covalently linked can lead to the formation of dead-end monomeric or homo-oligomeric complexes ranging from discrete oligomers to fibers^30^. Formation of such structures could compete with assembly of the desired triangle by serving as kinetic traps. To avoid this, we excluded designs composed of linked SYNZIPs for which even weak hetero-interactions were reported. After exclusion of these candidates, we selected a promising design composed of three chains of linked-SYNZIP fusion proteins: **1**–**6**, **5**–**4**, and **3**–**2** (Fig. 1D; a hyphen indicates a genetic fusion of two SYNZIP chains).

Once this promising design was selected, we used a short, flexible protein segment ((Gly_4_Ser)_2_) to link each pair of SYNZIPs, and included an affinity purification tag (His_6_) at the C-terminus. Plasmids encoding each fusion protein were expressed separately in *Escherichia coli*, followed by purification using nickel-affinity chromatography under denaturing conditions (Fig. S3).

### Self-assembly via thermal annealing

The separately prepared linked-SYNZIP fusion proteins were assembled into the designed heterotrimeric complex through thermal annealing. We devised a protocol to control the association of the three SYNZIP pairs via temperature-controlled cooling based on the reported melting temperatures (which are 47, 42 and 32 °C for **1**:**2**, **3**:**4**, and **5**:**6**, respectively)^20^. After unfolding at 95 °C, mixtures of fusion proteins were cooled and incubated at 42 °C for 1 hr, followed by rapid cooling to 4 °C (Fig. 1E). This two-step process was intended to allow desired pairings to occur at 42 °C before lowering the temperature to 4 °C, where some undesired complexes might be kinetically trapped.

**Figure 2:**
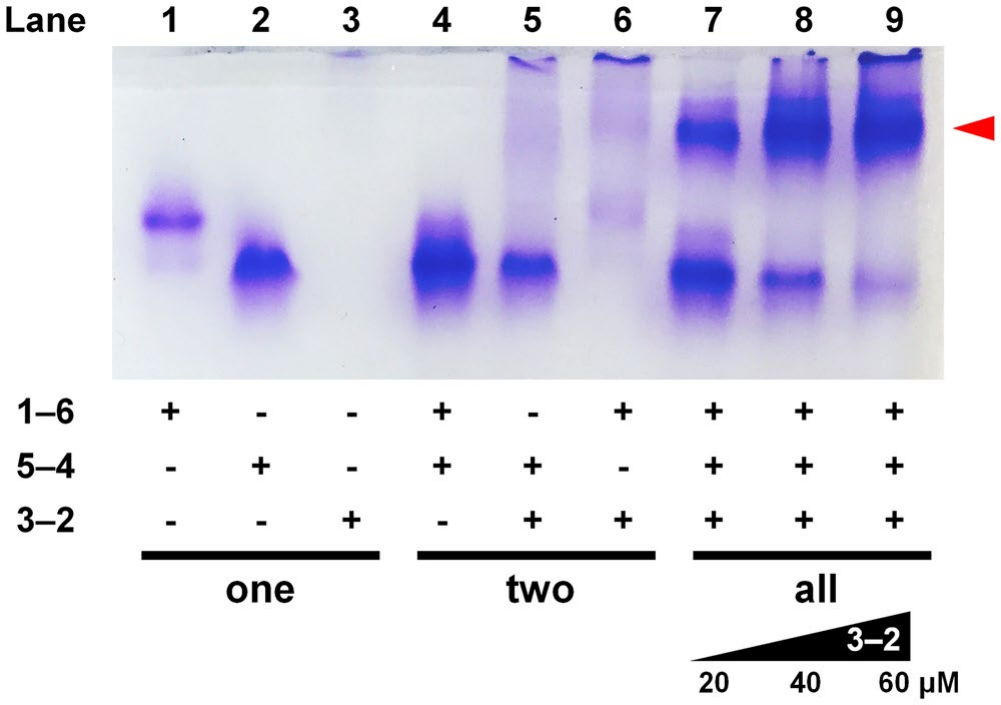
Native-PAGE analysis of interactions between linked-SYNZIP fusion proteins after thermal annealing. For lanes 1–6, one or two of constructs **1**–**6, 5**–**4** and/or **3**–**2** (20 µM each) were mixed and annealed. For lanes 7–9, **1–6** and **5–4** were mixed and annealed with varying concentrations of **3–2** (20, 40, or 60 µM). The red arrow indicates the band corresponding to the triangle.

**Figure 3:**
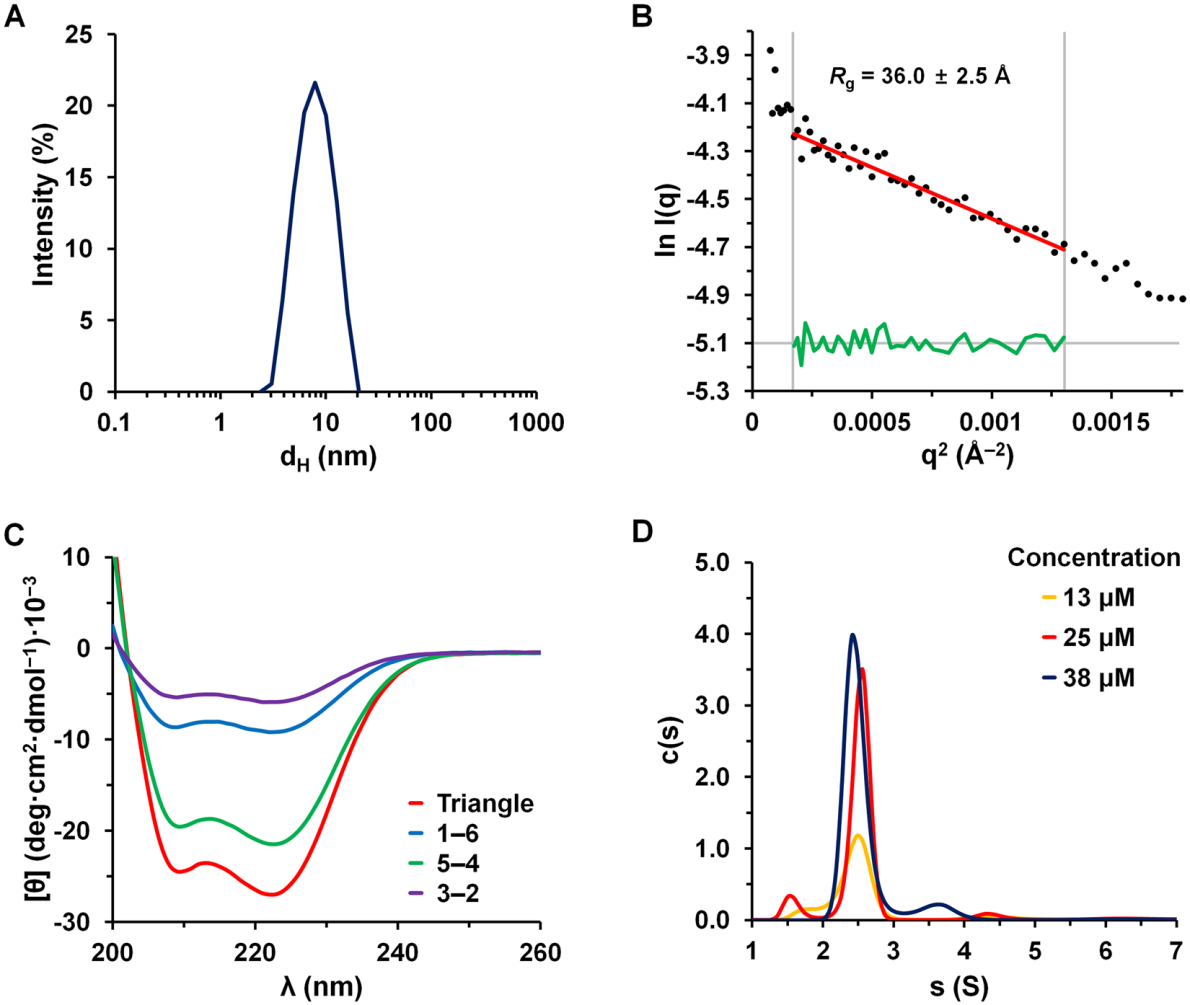
Characterization of size, folding, and assembly of the protein nanotriangle. (**A**) The hydrodynamic diameter (*d*_H_) of the protein nanotriangle as determined by DLS. (**B**) Guinier plot to determine *R*_g_ from SAXS measurements at 44 μM (black dots; Fig. S8). The fit (red line with residuals in green) is for data in the low-*q* range (*q* × *R*_g_ < 1.3). (**C**) CD spectra for **1**–**6**, **5**–**4**, and **3**–**2** and the purified assembly (triangle) (5 μM of each protein and 15 μM total for mixtures, 20 °C). (**D**) Distributions of sedimentation coefficient (s), in Svedbergs (S), for the protein nanotriangle at concentrations of 13, 25, and 38 μM, estimated from interference boundary fits (Fig. S11).

**Figure 4:**
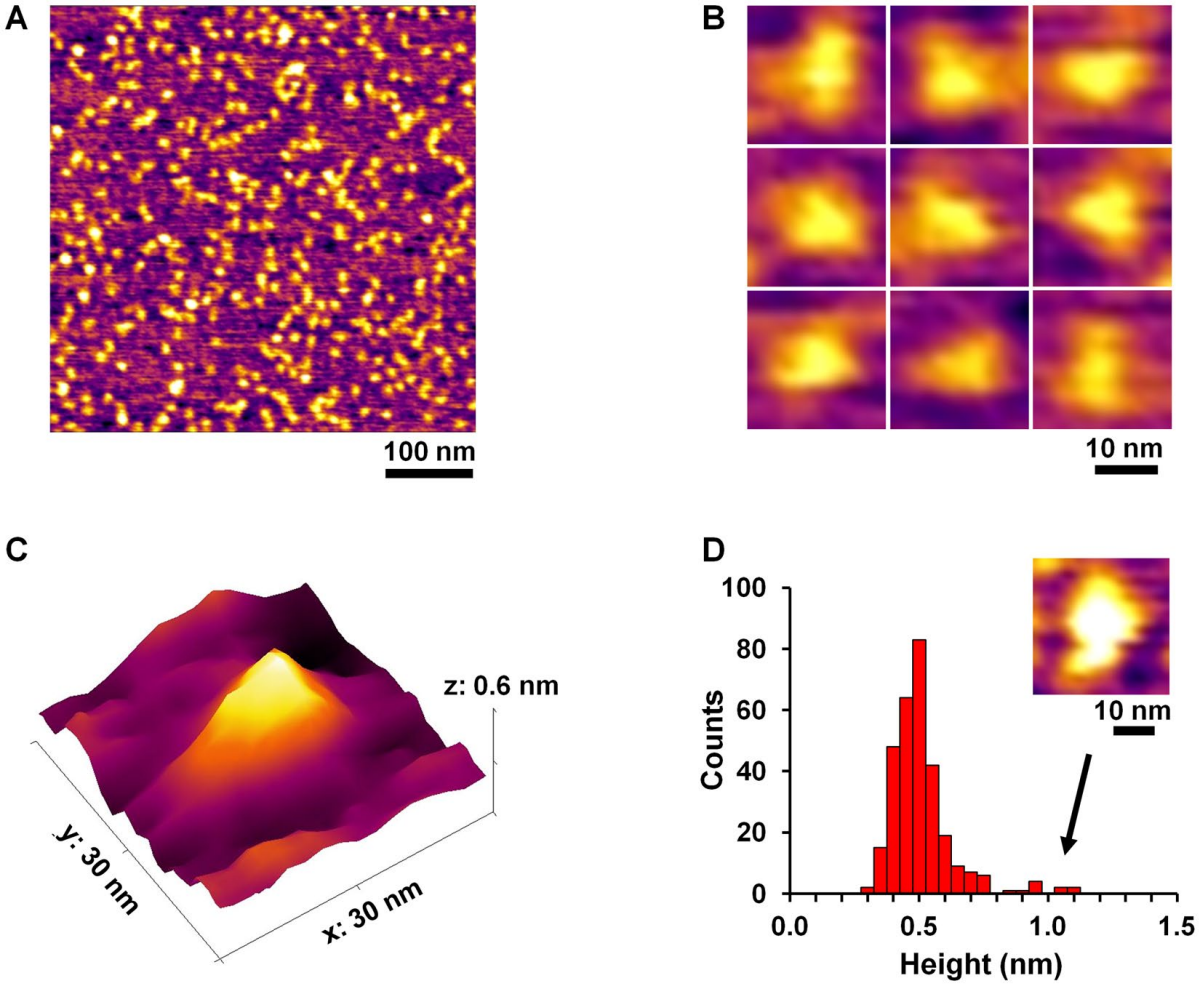
Characterization of the shape of the protein nanotriangle by AFM imaging. Height images of the triangles at low (**A**) and high magnifications (**B**), and a three-dimensional AFM image of a single assembly (**C**). (**D**) A histogram of the heights of the particles shown in panel A, and an image of a “tall” particle with height of ~1 nm (inset).

**Figure 5:**
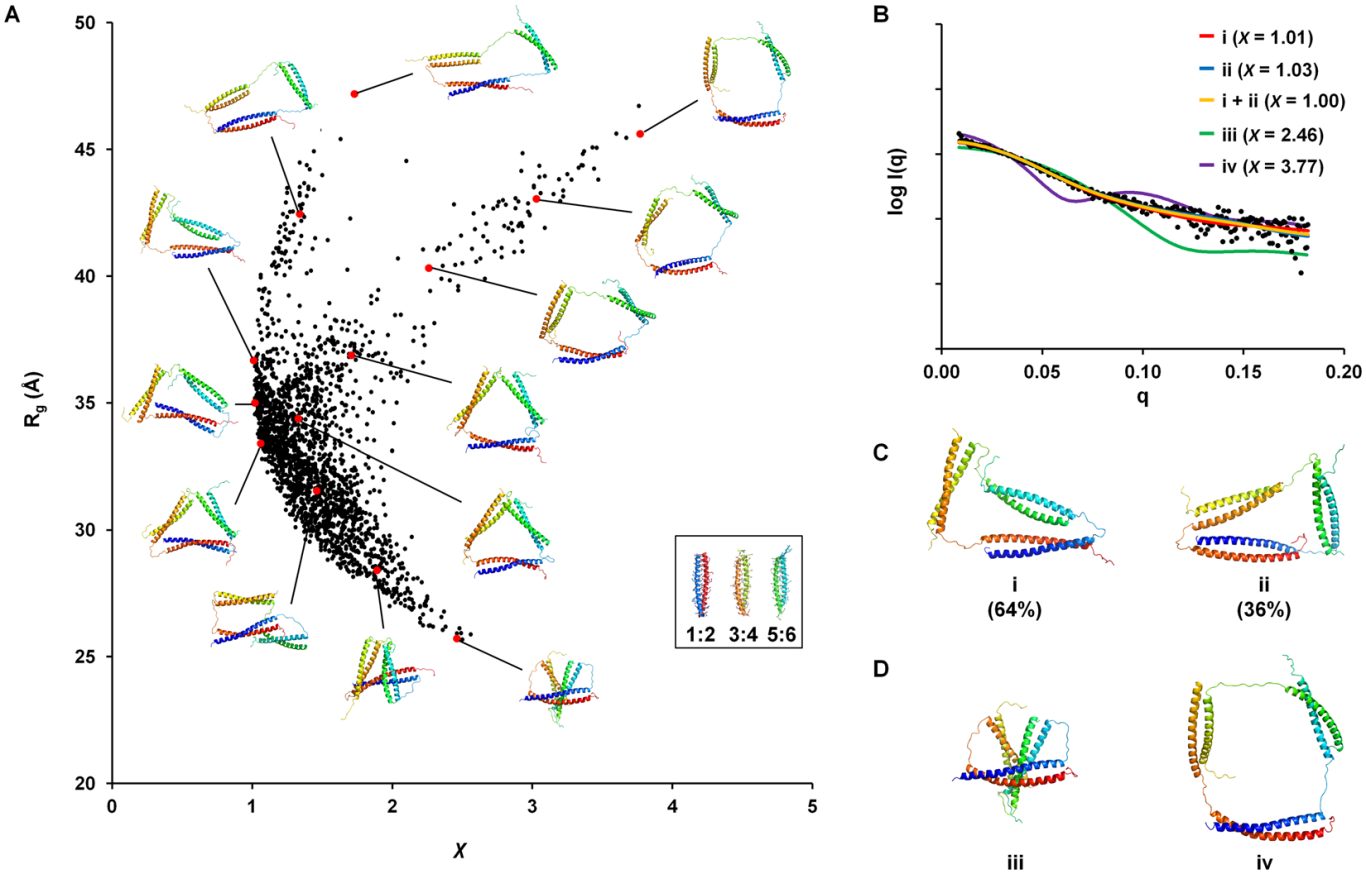
Structural models of the trimeric assembly and their agreement with experimental SAXS data. (**A**) R_g_ values for 1996 models plotted against χ values. Structures for representative models are shown next to the corresponding red dots. (**B**) Experimental data (black dots) and single-conformation or ensemble fits for structure models shown in panels C and D (colored lines). (**C**) Models with low χ values (*i* and *ii*) and the weights that define the ensemble in panel C. (**D**) Models with high χ values (*iii* and *iv*).

As confirmed by polyacrylamide gel electrophoresis under non-denaturing conditions (native PAGE), the fusion proteins formed a complex only when all three components required for the designed heterotrimer were present (Fig. 2). We observed a new band (indicated by the arrowhead) in which proteins migrated at a rate distinct from control samples in which only one (lane 1–3) or two (lane 4–6) components were included. This result supports formation of a protein complex composed of all the three linked SYNZIPs. After isolation using size-exclusion chromatography (SEC) (Fig. S4), we confirmed by mass spectrometry that the protein complex formed contained all three of the fusion proteins (Fig. S5). The isolated protein complex was stable for at least months at 4 °C and at submicromolar protein concentrations, as confirmed by biophysical characterization conducted after purification (Figs 3–5).

According to the calculated net charges of the linked SYNZIPs (Table S2), **3**–**2** is predicted to carry a charge of +2.6 at pH 7.4. This explains why a protein band is not observed for this species in electrophoresis of negatively charged species (Fig. 2, lane 3). Also, we observed that **3**–**2** was poorly soluble and prone to aggregation, which could also contribute to poor mobility in the gel. The assembly of **3**–**2** and **1**–**6** might also be insoluble (Fig. 2, lane 6), whereas the protein band that corresponds to **5**–**4** (lane 5) indicates that **3**–**2** and **5**–**4** may not associate efficiently at an equimolar mixing ratio under these conditions. Based on the observation that excess **3**–**2** led to increased yields (lanes 8 and 9), we hypothesize that an aggregation process, which limits the availability of **3**-**2**, competes with assembly into the nanotriangle.

In a control experiment, we annealed protein solutions by cooling to 25 °C and then to 4 °C, or cooling directly to 4 °C. The results showed that under either schedule, more residual **5**–**4** remained (indicated by an arrow in Fig. S6). The fast cooling below 42 °C may favor folding of **5**–**4** that competes with formation of the desired intermediate, as illustrated in Fig. S7. These results are consistent with the pathway in Fig. 1E but don’t rule out alternatives.

### Characterization of size, folding, assembly, and shape

The protein triangle in solution was highly monodisperse with a hydrodynamic diameter (*d*_H_) of 8.25 ± 0.14 nm, as determined by dynamic light scattering (DLS) (Fig. 3A). Guinier analysis of small-angle X-ray scattering (SAXS) data was used to determine radius of gyration (*R*_g_) of 36.0 ± 2.5 Å (Fig. 3B). These measurements are consistent with the expected size of the nanotriangle, given that the SYNZIP coiled-coil edges have lengths of 6.7, 5.7, and 5.5 nm (Fig. 1B). The data also confirm that the assembly does not form aggregates.

The helicity of the folded protein triangle was quantified using circular dichroism (CD) spectroscopy. Peaks at 208 and 222 nm in the CD spectrum indicated a helical structure consistent with the designed coiled-coil edges (Fig. 3C). The helix content estimated from the mean residue ellipticity (θ_222_ = −27.0 × 10^3^ deg cm^2^ dmol^−1^) was 79%, which is consistent with the value of 81% calculated based on the design. To estimate the expected helicity, we used SYNZIP crystal structures^20^ and homology models (see Methods), and assumed that the linker regions connecting SYNZIPs do not adopt a helical conformation. Unfolding with heat showed a cooperative structural transition with a melting temperature (T_m_) of ~50 °C (Fig. S9), which is slightly higher than the T_m_ of 47 °C for **1**:**2** (42 °C for **3**:**4** and 32 °C for **5**:**6**)^20^. In contrast, each of the unmixed, annealed fusion proteins showed less ellipticity at 222 nm than the nanotriangle (Fig. 3C); the low solubility of **3**–**2** may contribute to the lower CD intensity for this protein. Interestingly, **5**–**4** showed evidence of a partially helical structure, θ_222_ = −21.4 × 10^3^ deg cm^2^ dmol^−1^, despite the fact that interactions were not previously reported between SYNZIPs **5** and **4**. A helical structure is apparently populated by joining these sequences in the same chain. Although we did not perform detailed characterization of folded **5**–**4**, SEC experiments indicate that this protein elutes as expected for a dimer that is mixed with, or exchanging with, a smaller population of monomer (Fig. S10).

The molecular weight determined by analytical ultracentrifugation (AUC) was consistent with the value expected for the designed heterotrimer. Sedimentation velocity analysis was performed on samples at concentrations of 13, 25, and 38 μM, and we calculated sedimentation coefficient distributions c(s)^33^. The average s value for peaks measured at different concentrations was 2.5 ± 0.1 S (Fig. 3D), corresponding to a molecular weight of 34.9 ± 1.0 kDa. The deviation from the value predicted based on protein sequences (35.8 kDa) was less than 3% (Table S3). We also determined the molecular weight from SAXS data using an analysis that makes no assumptions about shape (Fig. 3B). The method we employed defines and uses the volume of correlation, *V*_c_, a SAXS invariant derived from the scattered intensities^34^. The molecular weight determined using this approach was 32.6 kDa, which is within the reported error range of this method (~10%)^34^.

We used atomic force microscopy (AFM) to image the designed protein assembly. Height images showed monodisperse nano-objects (Fig. 4A), consistent with the *d*_H_ value determined by DLS (Fig. 3A). Imaged objects appeared triangular in close-up images (Fig. 4B), with an average height of 0.51 ± 0.12 nm (Fig. 4C and D). The unimodal distribution indicated that the nanotriangles were uniform in height and thus likely to be discrete single particles (Fig. 4D). We observed a few tall particles with heights of ~1 nm, which we assumed to be aggregates or overlapped nanotriangles (see Fig. 4D inset). The average height for imaged particles was close to the previously measured height of a dimeric coiled coil on a mica substrate (~0.6 nm)^35^.

### Structural modeling with SAXS data

To further investigate the conformation of the protein nanotriangle in solution, we built all-atom models representing many different conformations and tested which models were consistent with the SAXS data. Using the crystal structures of **1**:**2** and **5**:**6** ^20^ and the homology model of **3**:**4**, we built models of the heterotrimer as described in the methods and, for each model, compared the predicted SAXS profiles for that structure to the experimental data^36^. We assessed agreement using the χ value, which has a value of 1.0 for models that fit the data within the accuracy of the experimental noise and larger values for less-good fits. Among 1996 diverse comparative models, which included highly collapsed and maximally expanded arrangements of coiled coils, the distribution of *R*_g_ values ranged from 26 to 47 Å, and χ values ranged from 1.01 to 3.77 (see Figs 5A and S12). Guinier analysis of the SAXS data gave *R*_g_ = 36.0 ± 2.5 Å (Fig. 3B), and it is apparent in Fig. S12 that most of the models with χ ≤ 1.05 had *R*_g_ values consistent with that range of values. Fig. 5C shows two models (*i* and *ii*) that are an excellent match to the data, with χ values of 1.01 and 1.03 and *R*_g_ values of 36.7 and 35.2 Å, respectively (Fig. 5B). In contrast, Fig. 5D shows models (*iii* and *iv*) with extreme values of *R*_g_ that had high χ values (2.46 and 3.77) and were clearly not consistent with the experimental SAXS profile shown in Fig. 5B. Given the flexible linkers used in the design, the triangle structure is best described as an ensemble of many different conformations. A two-structure ensemble consisting of 64% of model *i* and 36% of model *ii* agrees with the experimental observations within the noise level. Many other ensembles would also be consistent with the SAXS observations. However, our analysis rules out high occupancy of extremely compact structures in which the coiled-coil units themselves are strongly associating (as in Fig. 5D, *iii*), and also shows that the ensemble is not composed exclusively of highly expanded structures (Fig. 5A).

## Discussion

We designed and characterized a monodisperse, two-dimensional protein nanotriangle with a characteristic dimension of ~10 nm. To our knowledge, this is the first confirmed design of a protein structure with this topology. In prior work^30^, supramolecular assemblies were designed using a single type of heterodimeric coiled coil. Linkers of variable lengths were used to control the mode of assembly, from fibers to discrete nanoscale objects. One of the designs was predicted to adopt a triangular shape, although its shape was not directly characterized^30^.

In our simple strategy, self-assembly of a protein nanostructure was directed by the modularity of protein association, and a triangle structure was formed efficiently using a mixing and annealing procedure. Although we encountered some obstacles, such as unexpected folding of **5**–**4**, and limited solubility of unassembled proteins, these were overcome by modulating the annealing process and increasing the relative concentration of the poorly soluble component **3**–**2**. Flexibility in mixing and annealing procedures are advantages of our approach that here allowed us to realize a successful design. Our strategy has other advantages compared to alternatives for supramolecular assembly. For example, folding of a single protein chain into a tetrahedron, in prior work, was directed by six different coiled-coil modules, with the structure encoded in the ordered arrangement of sequences in the protein chain^29^. The fact that intra- over intermolecular interaction is favored at high dilution allowed for the formation of a complex structure. Micromolar concentrations lead to aggregation, as expected due to the propensity for self-association that is part of the design, and the strategy is not easily generalized. The design of cages or arrays using globular oligomeric domains requires symmetry matching, along with geometrical alignment of the domains using rigid^8–10^ or flexible linkers with optimal spacing^11, 13^. This is non-trivial to accomplish, and small differences can lead to the wrong stoichiometry^9^ or an infinite rather than finite assembly^30^. Finally, for some applications, it may be advantageous to trigger assembly at a specific time; in our scheme, this can be accomplished by mixing the components at a controlled time.

In this work, the pairwise interactions of three parallel, heterodimeric SYNZIPs were sufficient to direct supramolecular assembly. 23 SYNZIPs have been described^19, 20^, and binding pairs such as **10**:**22** and **17**:**18** could be useful as additional modules because their interactions are reported as stronger than potentially competing associations with any of SYNZIPs 1–6. Furthermore, synthetic coiled coils beyond SYNZIPs, as well as large sets of coiled - coil homo- and heterodimers from animal bZIP transcription factors that have been comprehensively tested for associations provide candidates for extended sets of orthogonal coiled coils^17, 18, 23, 37, 38^. With larger sets of coiled-coil modules that display strong interaction preferences, our approach may be extended to design more complex structures that can potentially be linked together in 2- or 3-dimensions, functionalized, attached to surfaces or incorporated with other designed elements into increasingly complex nanoassemblies.

## Methods

### Design of a protein nanotriangle

We designed a protein nanotriangle assembled from three chains of genetically linked SYNZIPs^19–21^. There are 16 candidate designs of this type that differ in which SYNZIPs are connected (Fig. S2A). We excluded designs that linked hetero-associating SYNZIPs, because such constructs could form complexes such as homo-oligomers and/or fibers that would compete with triangle assembly^30^. SYNZIP pairs **4**:**6**, **3**:**5**, **3**:**6**, and **1**:**5** are also known to interact weakly (Fig. 1A). Although the affinities of these pairs are orders of magnitude lower than the binding pairs (**1**:**2**, **3**:**4**, and **5**:**6**), the interactions may become significant when protein chains are linked together, increasing effective concentrations and potentially leading to kinetic traps. For example, linked-SYNZIP fusion proteins **4**–**6** or **3**–**5** can potentially form homodimers, with remaining coiled-coil segments forming heterodimers such as **5**–**2**/**1**–**3** (or **4**–**2**/**1**–**6)**; Fig. S2B illustrates some of these possibilities. We therefore excluded designs containing **3**–**5, 5**–**3, 4**–**6, 6**–**4, 3**–**6, 6**–**3, 1**–**5**, or **5**–**1**. Among the remaining four designs (in the top row of Fig. S2A), we chose to make the three chains **1**–**6**, **5**–**4**, and **3**–**2**. A flexible linker of tetraglycine-serine repeats (Gly_4_Ser)_2_ was placed between SYNZIPs, and an affinity purification tag (His_6_) was placed at the C-terminus of each polypeptide (Fig. 1D). The final sequences are given in Table S2.

### Construction of plasmids

We used standard molecular biology techniques to construct three plasmids that express the linked-SYNZIP fusion proteins. The genes encoding SYNZIPs were PCR-amplified using forward and reverse primers listed in Table S1. The amplified fragments were assembled into the designed DNA constructs, which were further amplified by PCR. After digestion by the restriction enzymes *NdeI* and *XhoI* (New England Biolabs), the inserts were ligated into an expression vector, pET-43.1a (Novagen). The resulting plasmids were sequenced to confirm insertion. Plasmids encoding individual SYNZIPs are available via AddGene (www.addgene.org).

### Protein expression and purification

Plasmids were separately transformed into *E. coli* strain BL21(DE3) cells (Agilent). Cell cultures for each fusion protein (1 L) were grown at 37 °C in Luria-Bertani liquid medium containing ampicillin (100 mg/L). At an optical density at 600 nm (OD_600_) of 0.6, isopropyl β-D-1-thiogalactopyranoside (IPTG) was added to induce protein expression (final concentration 1.0 mM). After 5 hours at 37 °C, cells were harvested by centrifugation. The harvested cells were resuspended in lysis buffer (8 M urea, 10 mM Tris-HCl, and 100 mM Na_2_HPO_4_ pH 8.0) and lysed by a cycle of freezing-thawing and sonication. The cell lysate was cleared by centrifugation, and incubated with nickel-nitrilotriacetic acid resin (Qiagen). In buffers containing 8 M urea, 10 mM Tris-HCl, and 100 mM Na_2_HPO_4_, the fusion proteins were washed at pH 6.3 and collected by elution at pH 4.5. The purified proteins were dialyzed into deionized water.

### Size-exclusion chromatography (SEC)

The annealed mixture of linked-SYNZIP fusion proteins was prepared at a total protein concentration of 100 µM (molar ratio of **1**–**6**, **5**–**4**, and **3**–**2** was 1:1:3) in 50 mM Tris-HCl pH 7.4, 150 mM NaCl. Samples (10 mL) were run on a fast protein liquid chromatography system over a Superdex 75 26/60 column (GE Healthcare) at a flow rate of 0.5 mL/min. Purity was confirmed by native-polyacrylamide gel electrophoresis (Native-PAGE).

### Circular dichroism (CD) spectroscopy

CD spectra were recorded on an AVIV 420 spectropolarimeter (Aviv Biomedical, Inc.). Protein solution samples were prepared in 50 mM Tris-HCl pH 7.4, 150 mM NaCl, 1 mM ethylenediaminetetraacetic acid (EDTA). Measurement was performed at 5 μM of each protein (15 μM total protein) in a 0.1-cm-length cuvette. The spectra were obtained at 20 °C in 1 nm increments in a wavelength range from 200 to 260 nm, averaging for 30 s at each wavelength. The α-helical content was estimated from the mean residual ellipticity at 222 nm, using the following equation^39^:

$${\rm{H}}{\rm{e}}{\rm{l}}{\rm{i}}{\rm{c}}{\rm{a}}{\rm{l}}\,{\rm{c}}{\rm{o}}{\rm{n}}{\rm{t}}{\rm{e}}{\rm{n}}{\rm{t}}\,({\rm{ \% }})=({[\theta ]}_{{\rm{o}}{\rm{b}}{\rm{s}}}{\rm{\times }}100)/\{{[\theta ]}_{{\rm{h}}{\rm{e}}{\rm{l}}{\rm{i}}{\rm{x}}}\times (1-2.57/n)\}$$

where [θ]_obs_ and [θ]_helix_ are the ellipticities of a helix of *n* and infinite residues, respectively. We used a [θ]_helix_ value of − 34,546 deg·cm^2^·dmol^−1^, which we computed based on the CD signal for SYNZIP1:SYNZIP2 and the number of helical residues observed in crystal structure (PDB ID: 3HE5)^20^. The thermal unfolding curve was determined by measuring ellipticity at 222 nm from 10 to 80 °C in 2 °C steps, with an averaging time of 30 s and an equilibration time of 1.5 min.

### Dynamic light scattering (DLS)

The hydrodynamic diameter of the protein nanotriangle was measured on a DynaPro NanoStar (Wyatt Technology), with a laser operating at a wavelength of 658 nm and at a detection angle of 90°. Protein solution (100 μl) was prepared in a cuvette, and measurements were performed at 25 °C. The raw correlation data were processed to generate a size distribution using DYNAMICS Software (Wyatt Technology).

### Analytical ultracentrifugation (AUC)

Sedimentation velocity AUC was performed using a Beckman XL-I centrifuge with interference optics. Protein samples were prepared at concentrations of 13, 25, and 38 μM in 50 mM Tris-HCl pH 7.4, 150 mM NaCl, and 1 mM EDTA. The samples were dialyzed against the same buffer overnight. All measurements were made at 20 °C with a rotor speed of 42,000 rpm. Data were analyzed using SEDFIT (http://www.analyticalultracentrifugation.com)^33^, and sedimentation coefficient distributions c(s) were obtained. Parameters required for data analysis (protein partial specific volume, buffer viscosity, and buffer density) were calculated using the SEDNTERP web server (Biomolecular Interaction Technologies Center, http://sednterp.unh.edu).

### Atomic force microscopy (AFM)

Freshly cleaved mica was incubated with poly-L-lysine (Sigma, MW 1,000–5,000) dissolved in deionized water (0.015 w/v %) for 10 min. The protein nanotriangle in 50 mM Tris-HCl pH 7.4, 150 mM NaCl, and 1 mM EDTA, and at a concentration of 500 nM, was deposited onto the mica treated with poly-L-lysine for 1 min. The mica was washed with 1 mL of MilliQ-filtered deionized water and blown dry in a stream of nitrogen gas.

Imaging was carried out using the Asylum Research Cypher microscope at the Center for Nanoscale Systems (CNS) at Harvard University. Imaging was done in air for topography measurement, using tapping mode in the repulsive interaction regime. Tapping parameters were tuned such that the tip started tracking the surface in the attractive interactions regime, and then the strength of interactions was increased gradually, into the repulsive regime, at which point high-resolution AFM images were obtained. We used AFM tip AC240BSA-R3 from Asylum Research (f = 75 kHz, k = 2 N/m).

### Biological small-angle X-ray scattering (BioSAXS)

The protein nanotriangle solution samples were prepared in 50 mM Tris-HCl pH 7.4, 150 mM NaCl, and 1 mM EDTA, and at concentrations of 11, 22, and 44 μM. Synchrotron X-ray scattering data was collected at the G-line of the Cornell High Energy Synchrotron Source (G1)^40, 41^. Area Detector System CCD was used to collect the scattering patterns, and data analysis was done using BioXTAS RAW (version 1.0.0) to determine *R*_g_ and molecular weight.

### Modeling

A comparative protein structure model for **3**:**4** was built with MODELLER^31^, using structures of coiled coils (PDB ID: 1KD8, 1KD9^42^, and 3HE4^20^) as templates. Using this model as a starting structure, molecular dynamics (MD) simulations were performed using the NAMD 2.11 package^43^ and the CHARMM22 all-atom force field^44^. The protein nanotriangle was solvated within a 60.7 × 95.2 × 85.2 Å^3^ water box, using periodic boundary conditions. The system was simulated in the constant temperature and pressure ensemble at 298 K for over 10 ns.

Comparative structure models for the protein nanotriangle were built using MODELLER^31^ and fit to experimental SAXS data using the FoXS/MES web server software (https://modbase.compbio.ucsf.edu)^36^. As templates for modeling, we used x-ray structures for **1**:**2** and **5**:**6** (PDB ID: 3HE5 and 3HE4)^20^. For **3**:**4**, we used the two coiled-coil structures 1KD8 and 1KD9^42^. We confirmed that models of the coiled-coil part of **3**:**4**, in the context of the triangle models, agreed well with structures from the MD simulation (at 10 ns) shown in Fig. S1 (backbone atom RMSD < 3.5 Å for 1984 models). No spatial restraints were applied to the flexible linkers between SYNZIPs. Numerous models were generated by choosing different, random initial positions for the coiled coils in the starting templates, and then refining the models using molecular dynamics with simulated annealing. The end-to-end distance (d_linker_) of the 1996 resulting structures ranged from 3 to 33 Å. The 1996 resulting structures were fit to the SAXS data, and the quality of the fit was measured by the χ function^36^:

2 $$\chi =\sqrt{\frac{1}{M}\sum _{i=1}^{M}{(\frac{{I}_{\exp }({q}_{i})-cI({q}_{i})}{\sigma ({q}_{i})})}^{2}}$$

where M is the number of points in the SAXS profile, *I*_*exp*_ *(q*_*i*_) and *I(q*_*i*_) are the experimental and computed profiles, *c* is the scale factor, and *σ(q*_*i*_) is the experimental error that represents standard deviation of intensity values. Various combinations of models with χ ≤ 1.05 were selected and used to fit ensembles to the SAXS data using ensemble search software (MES)^45^. Scattering intensities of the multiple conformations of a minimal ensemble were computed by averaging individual scattering patterns.

## Electronic supplementary material

Supplementary Information
