# Supplementary material for: Modular assembly of a protein nanotriangle using orthogonally interacting coiled coils
Source: Sci Rep. 2017 Sep 5;7:10577. doi: 10.1038/s41598-017-10918-6 (PMC5585338; doi:10.1038/s41598-017-10918-6)
Supplement: Supplementary file 1 — Supplementary Information [file 41598_2017_10918_MOESM1_ESM.pdf]

Supplementary Information

Modular assembly of a protein nanotriangle using orthogonally interacting coiled coils

Won Min Park,[a] Mostafa Bedewy,[b,†] Karl K. Berggren,[b,c] Amy E. Keating[a,d,*]

[1]Department of Biology, [2]Research Laboratory of Electronics, [3]Department of Electrical Engineering and Computer Science, and [4]Department of Biological Engineering, Massachusetts Institute of Technology, 77 Massachusetts Avenue, Cambridge, MA 02139, United States

[†]Present Address: Department of Industrial Engineering, University of Pittsburgh, 3700 O'Hara Street, Pittsburgh, Pennsylvania 15261, United States

[*]Correspondence to: keating@mit.edu

Supplementary Figures

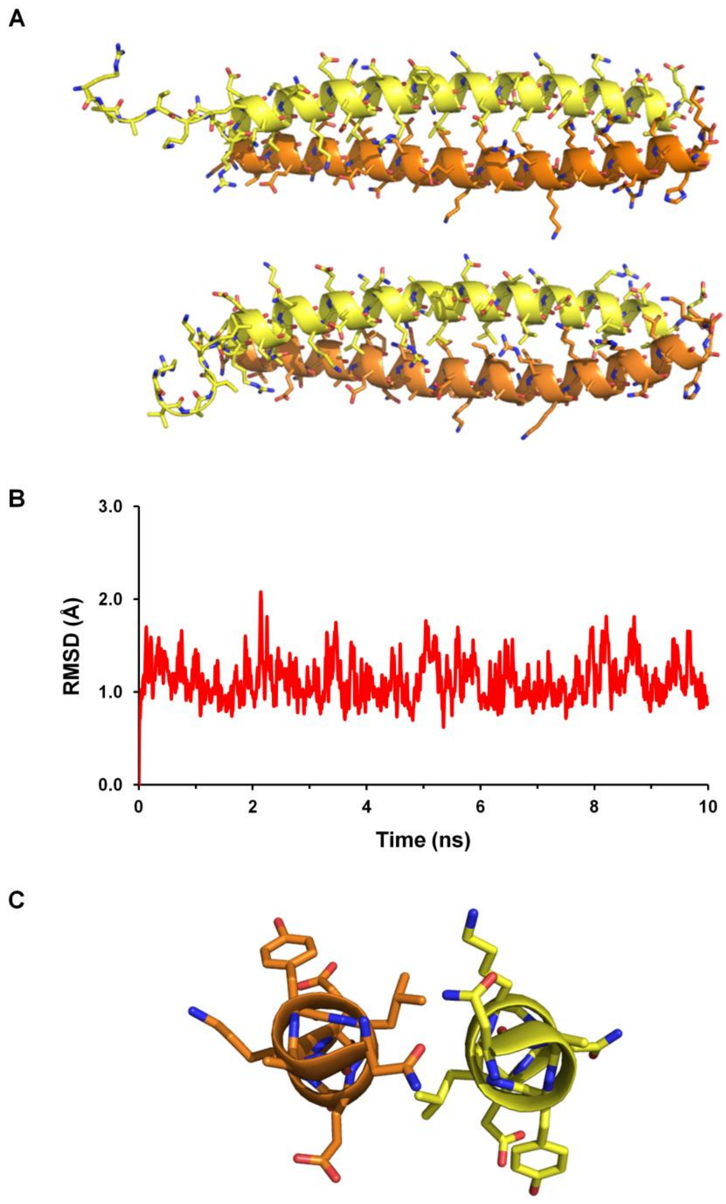

Figure S1. Structural model of a parallel dimer formed by **3** (orange) and **4** (yellow). (A) Models built from templates (top), and after 10 ns of molecular dynamics (MD) simulation (bottom). (B) The root-mean-squared deviation (RMSD) values relative to the starting structure for the coiled-coil backbone atoms (residues 1 – 42 of **3** and residues 15 – 54 of **4**) during MD simulations. (C) Cross section of the third heptad (residues 15 – 21 of **3** and residues 29 – 35 of **4**) after 10 ns of MD simulations, as viewed from the N-terminus.

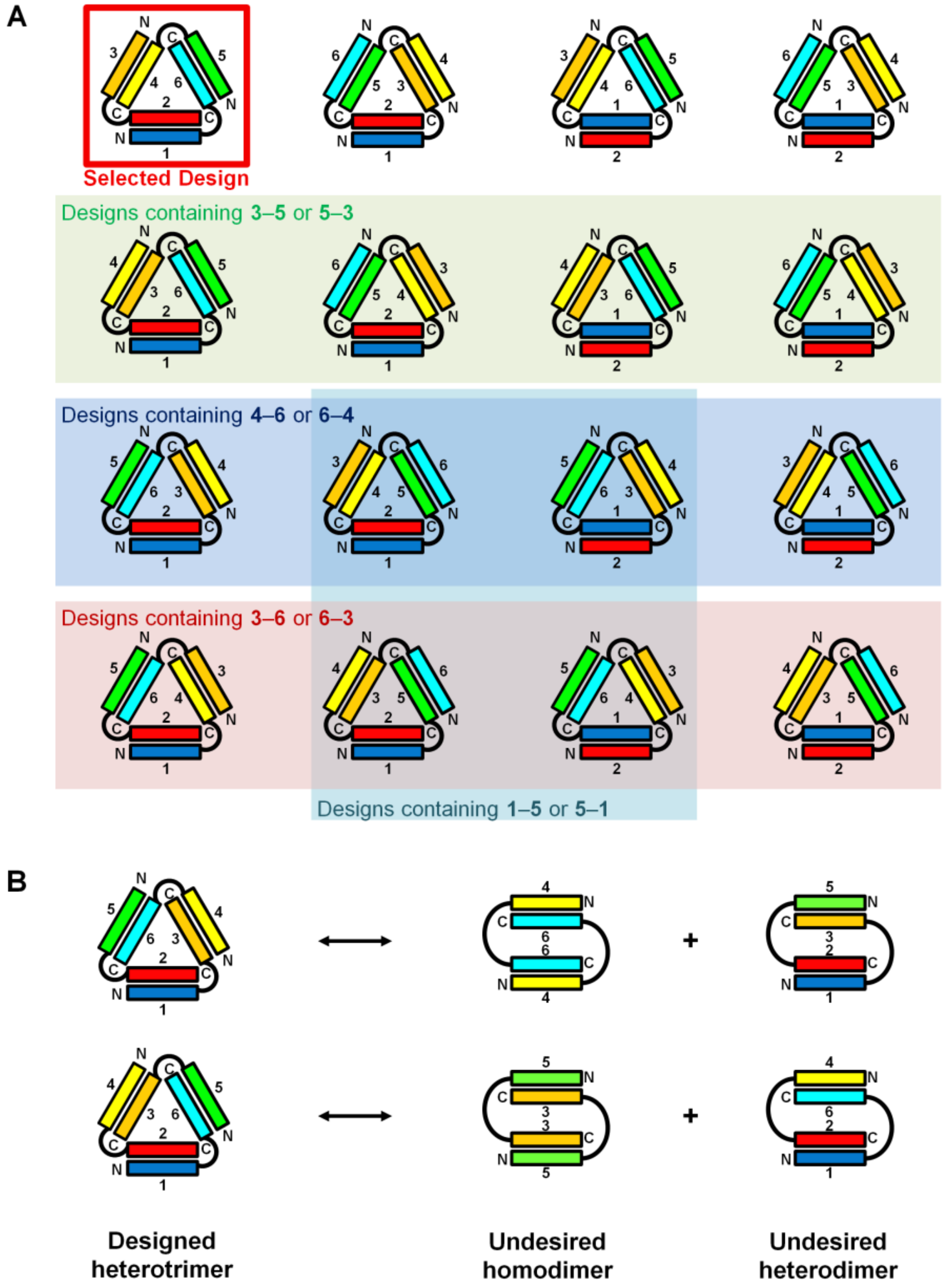

Figure S2. Design of a self-assembled protein nanotriangle. (A) 16 designs that combine three orthogonal SYNZIP pairs in a three-chain topology. The designs containing undesired combinations **3**–**5, 5**–**3, 4**–**6, 6**–**4, 3**–**6, 6**–**3, 1**–**5**, and **5**–**1** are shaded, and the selected design is indicated in a red box. (B) Illustration of how some designs could lead to formation of homodimers (**4**–**6**, top or **5**–**3**, bottom) and heterodimers, rather than the desired heterotrimer.

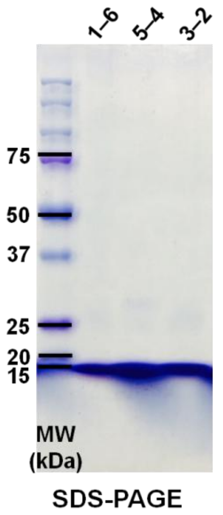

Figure S3. Biosynthesis of linked-SYNZIP fusion proteins. SDS-PAGE gel of **1**–**6**, **5**–**4**, and **3**–**2**, which have expected molecular weights of 11748, 11809, and 12197 Da, respectively.

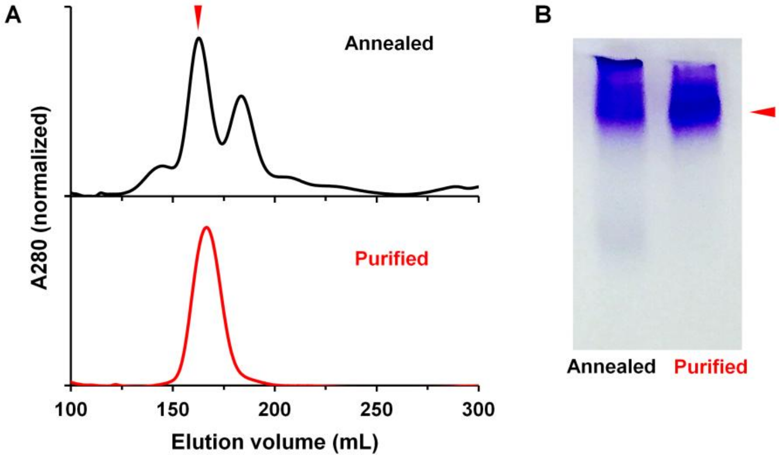

Figure S4. Purification of the protein nanotriangle. (A) Size-exclusion chromatography (SEC) profiles of the annealed mixture (black) and purified complex (red). (B) Native-PAGE gel of the annealed mixture (left) and purified complex (right). The position of the protein nanotriangle in the SEC profiles and native-PAGE gel are indicated by red arrowheads.

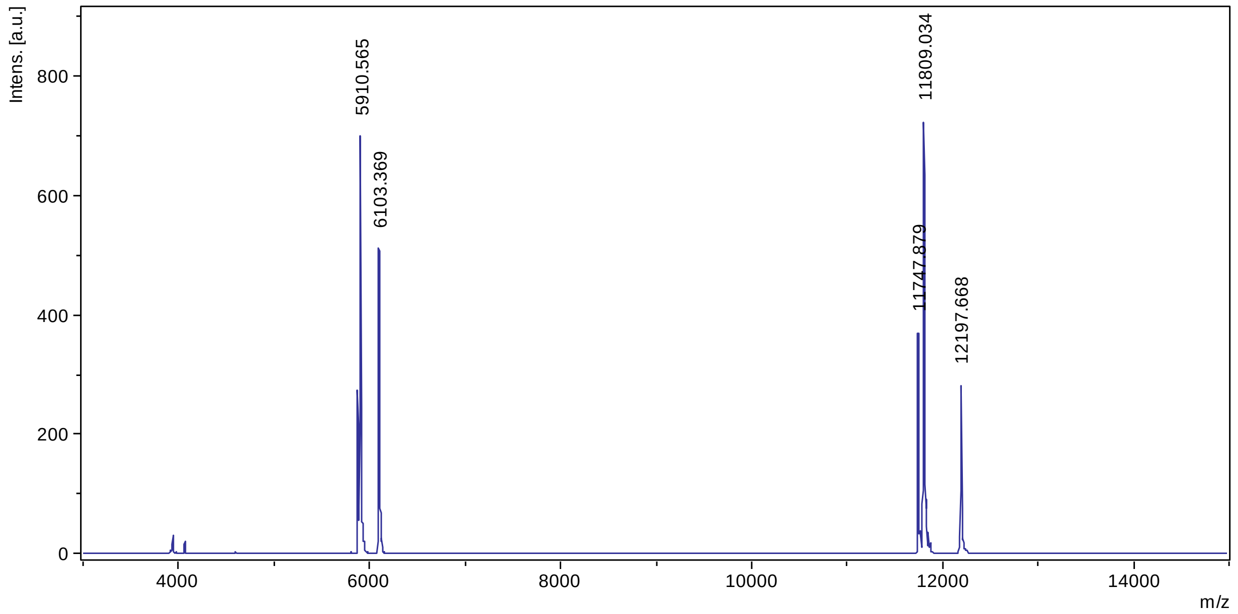

Figure S5. Mass spectrum of the protein nanotriangle. The peaks at m/z 11748.879, 11809.034, and 12198.668 correspond to the linked-SYNZIP fusion proteins. The theoretical molecular weights estimated from protein sequences of **1**–**6**, **5**–**4**, and **3**–**2** are 11748, 11809, and 12197 Da, respectively.

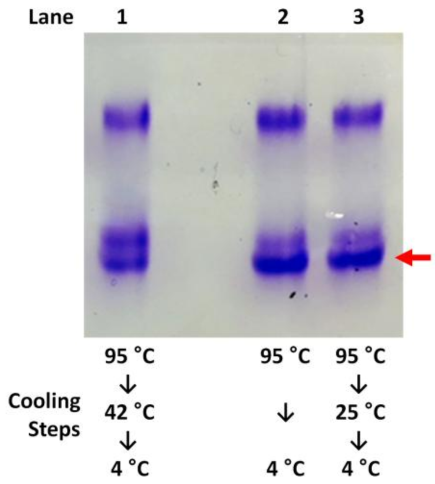

Figure S6. Native-PAGE analysis of interactions between linked SYNZIPs following different cooling protocols. After mixing and denaturing the peptides at 95 °C, protein solutions were cooled to 4 °C with different cooling schedules, as described below the gel image. Constructs **1**–**6**, **5**–**4** and **3**–**2** were mixed and annealed at 20 μM each. Red arrow indicates **5**–**4**.

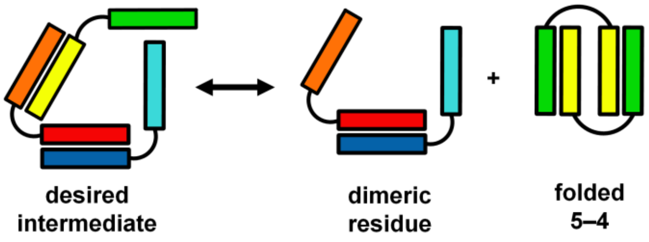

Figure S7. Scheme of the competition between folding of **5–4** into a hypothetical dimer and formation of the desired intermediate during fast cooling below 42 °C.

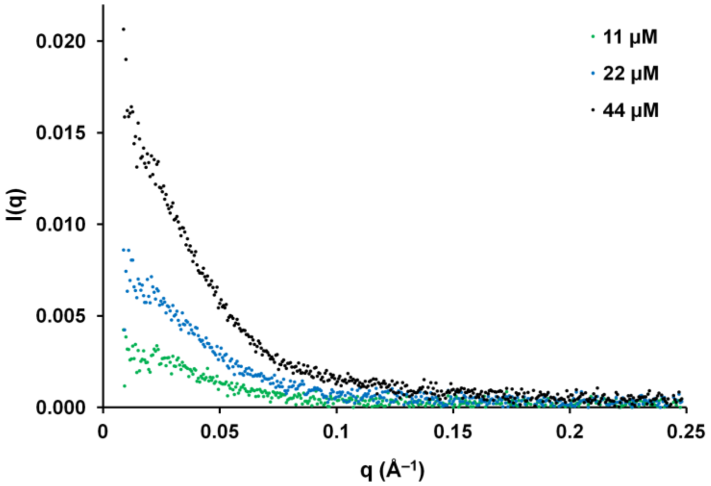

Figure S8. The scattering profiles of the protein nanotriangle at concentrations of 11, 22, and 44 µM.

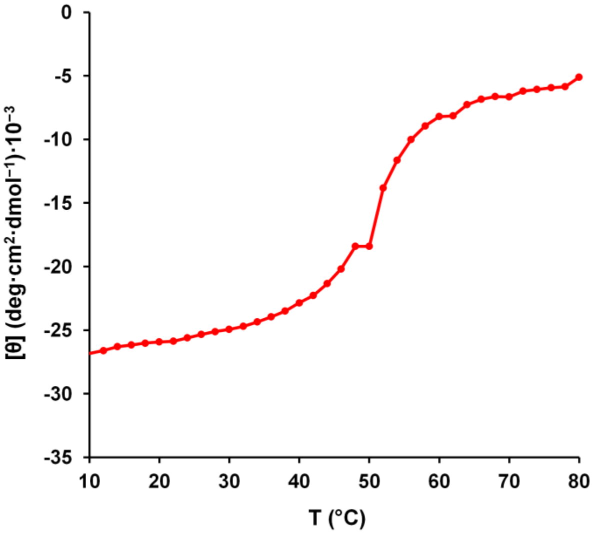

Figure S9. The melting curve of the protein nanotriangle as determined by circular dichroism at 222 nm. The purified protein nanotriangle was measured at a concentration of 5 μM of each protein (15 μM total protein concentration).

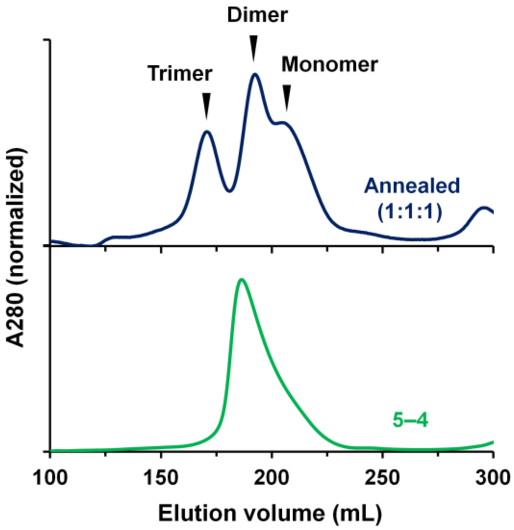

Figure S10. Linked SYNZIP **5**–**4** forms dimers by SEC. SEC profile of a mixture of **1**–**6**, **5**–**4** and **3**–**2** annealed at equilmolar ratios (1:1:1) (top) compared to linked SYNZIP **5**–**4** alone (bottom). Peaks for the trimer (~36 kDa), dimer (~24 kDa), and monomer (~12 kDa) of the linked-SYNZIPs are indicated by red arrowheads. The proteins were at concentrations of 60 μM (top, 20 μM for each protein) and 100 μM (bottom), respectively.

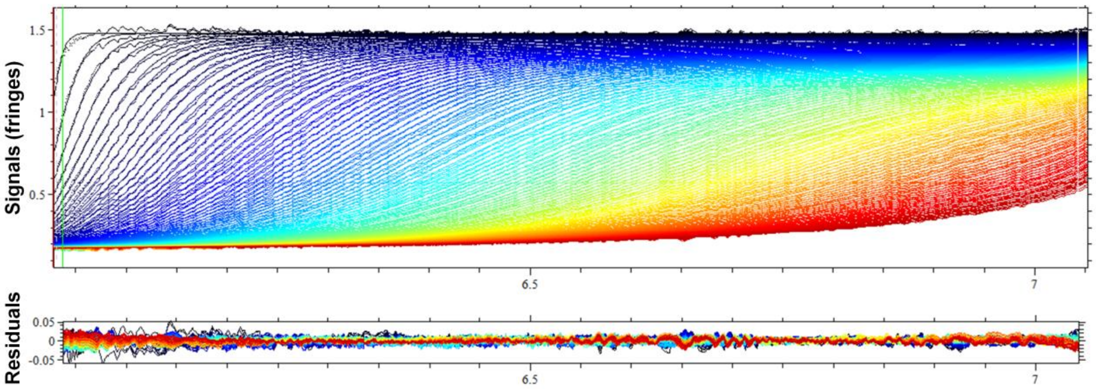

Figure S11. Velocity sedimentation data for the nanotriangle. Interference boundary data and fits (top), and the corresponding residuals (bottom), for the protein nanotriangle at a concentration of 25 μM.

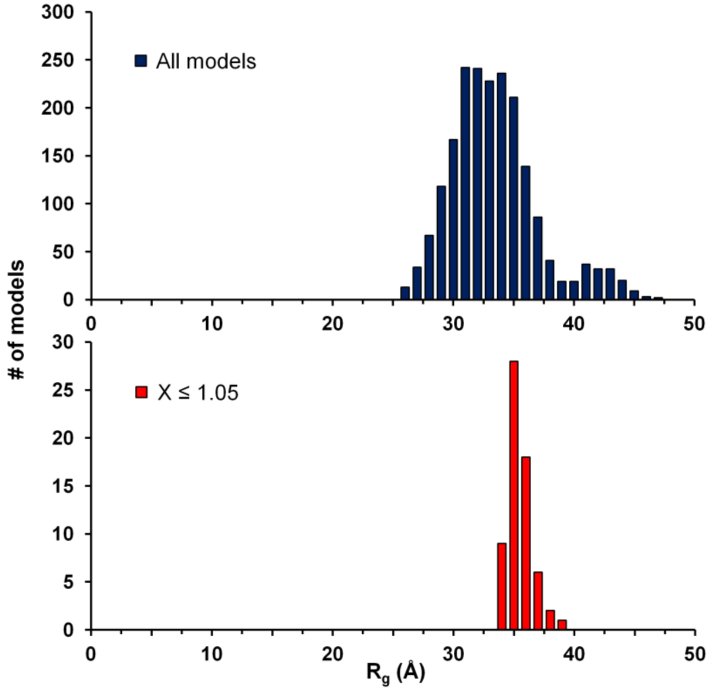

Figure S12. Distributions of R_g values calculated for the models built by comparative modeling: all 1996 models (top) and the models with $\chi \leq 1.05$ (bottom). The R_g value estimated from the SAXS Guinier analysis is 35.95 ± 2.52 Å (Figure 3B).

Supplementary Tables

Table S1. Sequences of primers used for PCR

Name	Sequence (5' to 3')
1_forward	TATCATCATATGAACCTGGTTGCGCAGCTCGAAAAC
1_reverse	CACCACCGCCAGAGCCACCACCGCCTTCGATTTTCTTACGCAGATTCGCGATTTC
2_ forward	GTGGTGGCTCTGGCGGTGGTGGATCCGCGCGTAACGCGTATCTGC
2_ reverse	TATCATCTCGAGCGCAACTTCGTTTTCGAGACG
3_ forward	AATCATCATATGAACGAAGTTACCACTCTGGAGAATG
3_ reverse	CACCACCGCCAGAGCCACCACCGCCTTTTTTGTGCGCCAGACGGTTG
4_ forward	GTGGTGGCTCTGGCGGTGGTGGATCCAATCGTAACGAACAGCTGAAAAACAAAG
4_ reverse	TATCATCTCGAGTTCTGCAACGTCGTTTTCCAGAC
5_ forward	TATCATCATATGAACACCGTTAAAGAACTGAAAAACTACATC
5_ reverse	CACCACCGCCAGAGCCACCACCGCCCTCGAATTTGTGAGCCGCCAG
6_ forward	GTGGTGGCTCTGGCGGTGGTGGATCCAAAGAAAACGCGAAGCTGGAGAAC
6_ reverse	TATCATCTCGAGACGCGCAACGTCACGTTCCAG

Table S2. Sequences of the linked-SYNZIP fusion proteins

Name	Sequence	pI	Net charge at pH 7.4
**1−6**	M**NLVAQLENEVASLENENETLKKKNLHKKDLIAYLEKEIAN LRKKIE**GGGGSGGGGS**KENAKLENIVARLENDNANLEKDI ANLEKDIANLERDVAR**LEHHHHHH	6.41	– 3.4
**5−4**	M**NTVKELKNYIQELEERNAELKNLKEHLKFAKAELEFELAA HKFE**GGGGSGGGGS**NRNEQLKNKVEELKNRNAYLKNELA TLENEVARLENDVAE**LEHHHHHH	6.32	– 4.3
**3−2**	M**NEVTTLENDAAFIENENAYLEKEIARLRKEKAALRNRLAH KK**GGGGSGGGGS**ARNAYLRKKIARLKKDNLQLERDEQNL EKIIANLRDEIARLENEVA**LEHHHHHH	9.02	+ 2.6

*SYNZIPs are colored: **1** (blue), **2** (red), **3** (orange), **4** (yellow), **5** (green), and **6** (sky blue).

** The pI and net charge values were calculated by the Protein Calculator v.3.4 (http://protcalc.sourceforge.net).

Table S3. AUC data

**Conc. (μM)**	***f/f₀***	**s (S)**	**MW by AUC (kDa)**	**MW error (%)**
38	1.48	2.47	34.7	2.9
25	1.48	2.53	35.9	0.5
13	1.50	2.40	33.9	5.1
Average	1.49 ± 0.01	2.47 ± 0.07	34.9 ± 1.0	2.5
